# The sedimentation datasets of Keuliling reservoir

**DOI:** 10.1016/j.dib.2020.106181

**Published:** 2020-08-19

**Authors:** Armido Armido, Azmeri Azmeri, Eldina Fatimah, Nurbaiti Nurbaiti, Silvya Norma Yolanda

**Affiliations:** a Department of Civil Engineering, Faculty of Engineering, Universitas Syiah Kuala, Indonesia; b Ministry of Public Works and Housing (PUPR) BWS Sumatera, Sumatera, Indonesia

**Keywords:** Sediment, Keuliling Reservoir, Measurement, Analyzed

## Abstract

Sedimentation is commonly found as one of the problems affecting the function of reservoirs in Indonesia. High risk soil erosion leads to the increase in sediment production which negatively influencing the capacity or functional age of the reservoir. Generally, several reservoirs in Indonesia are experiencing the operational problem due to the increasing of sedimentation throughout the year. The data presented in this article was obtained from the measurement of sediment in the area of Keuliling reservoir, including: The data of suspended sediment at every Keulling reservoir water inlet, the data of bedload sediment at the bottom of the Keuliling reservoir, the hydrometric data, including the water velocity and channel profile, where the sampling of bedload and suspended sediment sampling was conducted at each inlet. the data sediment volume at the dam reservoir. The data of suspended and bedload sediment were obtained by taking the samples and analyzed in a laboratory. The measurement was conducted three times in five months. The data of water velocity was also generated based on the measurement using a currentmeter. Meanwhile, the sediment volume data in the water storage of reservoir was gathered by conducting a bathymetry and topographic survey at the dam reservoir location and then comparing it with the pre-existing bathymetry and topographic data.

**Specifications Table**

**Table:** 

Subject	Civil Engineering and Water Resource Management
Type of data	Raw, Table, Image, Fig.
How data were acquired	Raw data obtained from the results of previous measurements will be analyzed using SMS software to get the volume and distribution of sediment.
Data format	Raw and Analysed
Parameters for data collection	The measurements of hydrometric, suspended sediment, and bedload sediment were conducted three times, in wet season condition and dry season condition. The bedload sediments in the Keuliling Reservoir are greyed-mud which predominantly comprises Fine with a diameter of <0.075 mm as well as a few Sand (> 0.075 mm and <2.0 mm).
Description of data collection	The volumetric flow rate measurement was conducted based on the Equal Width Increment Method, by dividing the width of the river cross section into several equal parts depending on the number of samples to be taken. The vertical sampling was located in the middle of the cross section of the sampling area. The sampling was conducted using the point sample method due to the shallow and narrow flow. In the measurement of bedload sediment transport, riverbed material samples are taken several times at different depths of flow in the same place.
Data source location	City/Town/Region: Aceh Besar, Aceh Country: Indonesia Latitude and longitude (and GPS coordinates) for collected samples/data: [see Appendix A of the Supplementary Data associated with this paper]
Data accessibility	With the article and in the following repository data. Repository name: Mendeley Data Data identifiacation number: DOI: xxx Direct URL to data: https:/Mendeley.com/datasets/xxxxx

**Value of the Data**

• The data of suspended and bedload sediment in each inlet of the Keuliling reservoir can be used to calculate the sedimentation rate entering the Keuliling reservoir, either by using formulas or numerically.
• The volume of sediment at Keuliling Reservoir can be used as a calibration of numerical sedimentation models in the Keuliling Reservoir
• The volume of sediment in the reservoir can be directly used as a reference for dredging the Keuliling Reservoir.

## 1 Data description

**Fig. 1:**
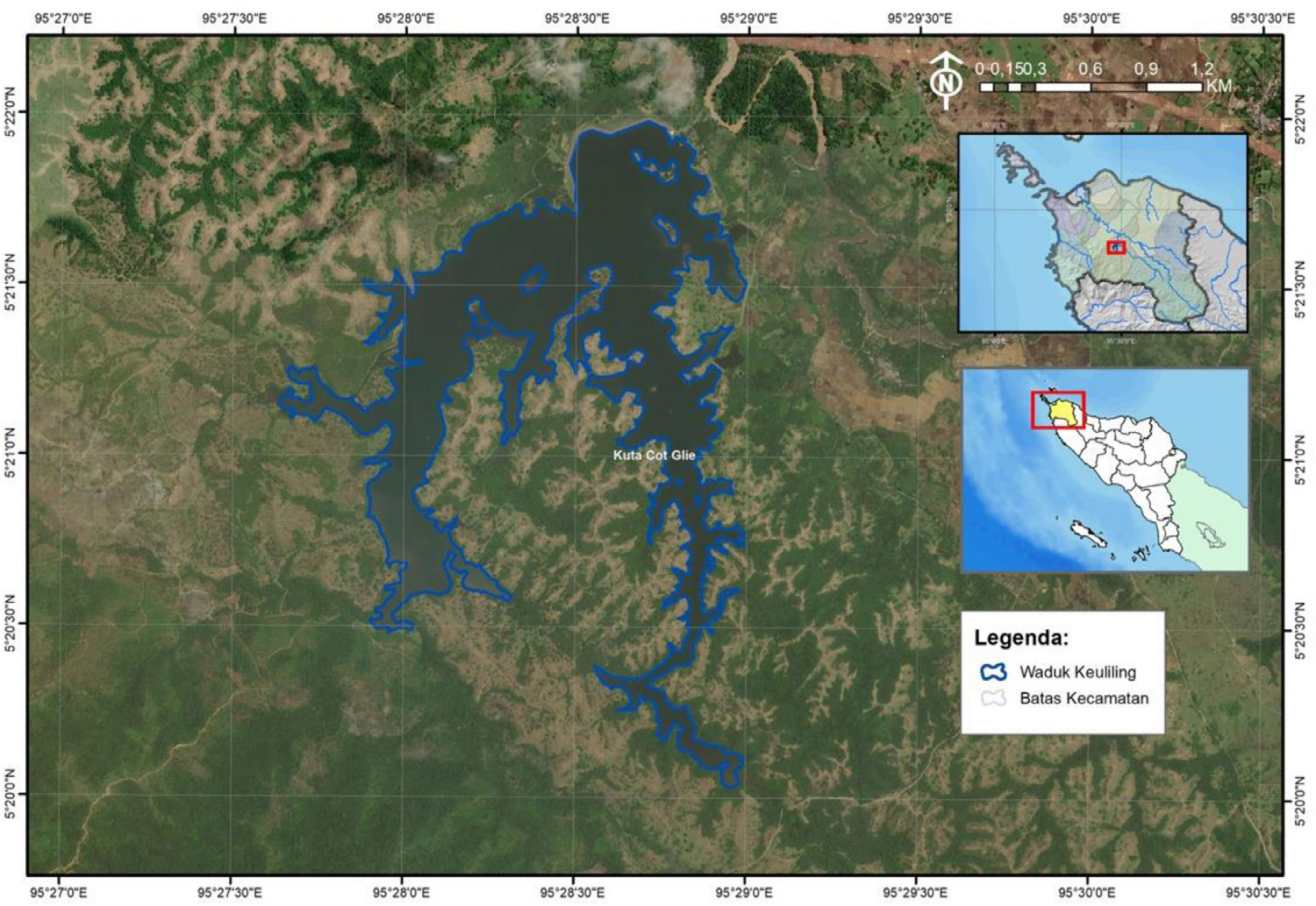
Location of The Study Area

The study was conducted at Keuliling Reservoir,Cot Glie District, Aceh Besar Regency,Aceh Province,Indonesia. It is located ±35 km from Banda Aceh. The location can be seen in Fig. 1.

**Fig. 2:**
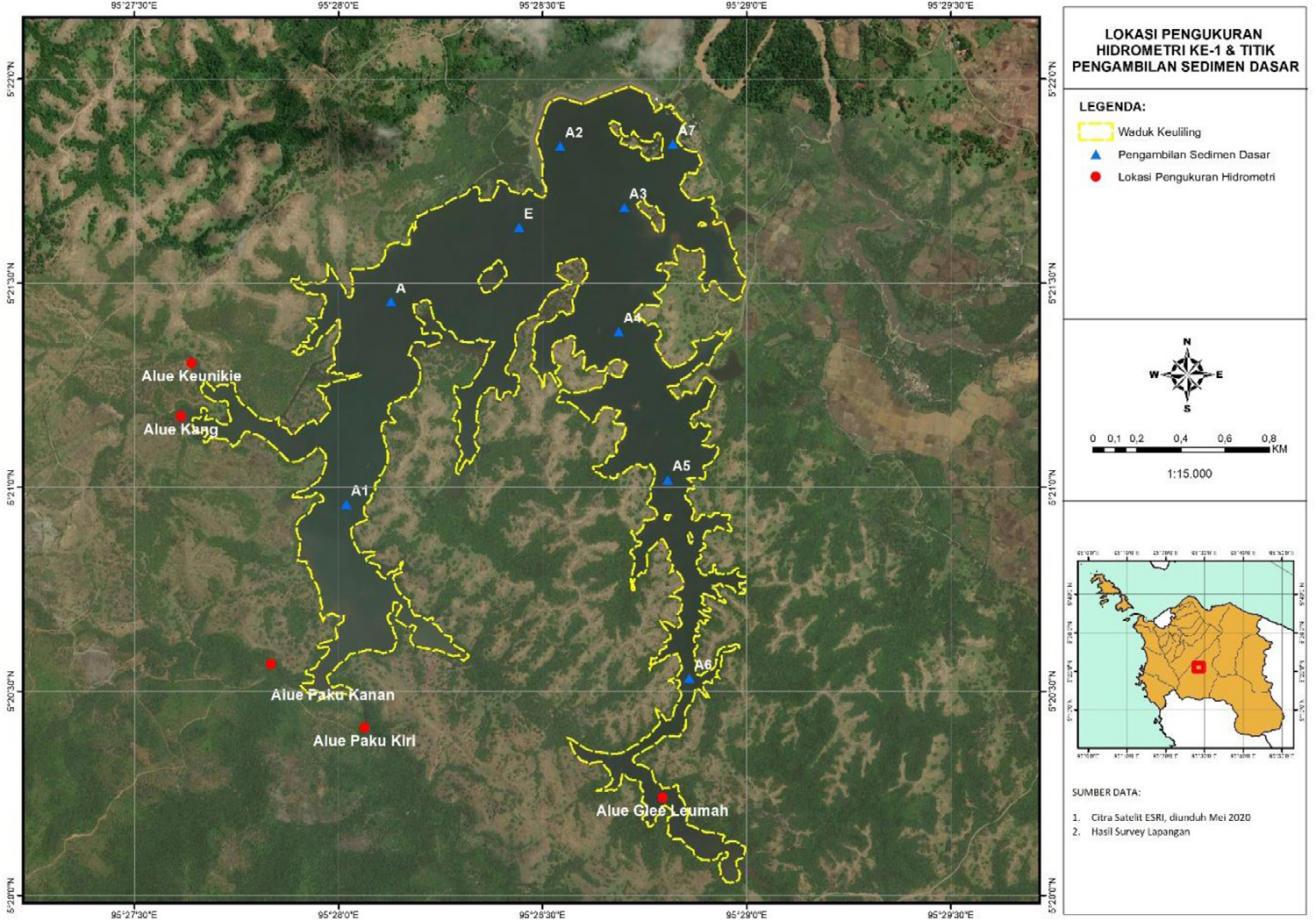
Location of hydrometric, suspended sediment and bedload sediment measurements

The measurements of hydrometric, suspended sediment, and bedload sediment were conducted three times, on 25 April 2018, 29 May 2018 (wet season condition) and 21 August 2018 (dry season condition) at five locations. Each location is the inlet of the Keuliling Reservoir, as shown in Fig. 2.

The measurement of bedload sediments was carried out in the nine water storages spreading in the area of Keuliling Reservoir.

## 2 Experimental Design, Materials And Methods

### 2.1 Hydrometric Measurement

**Fig. 3:**
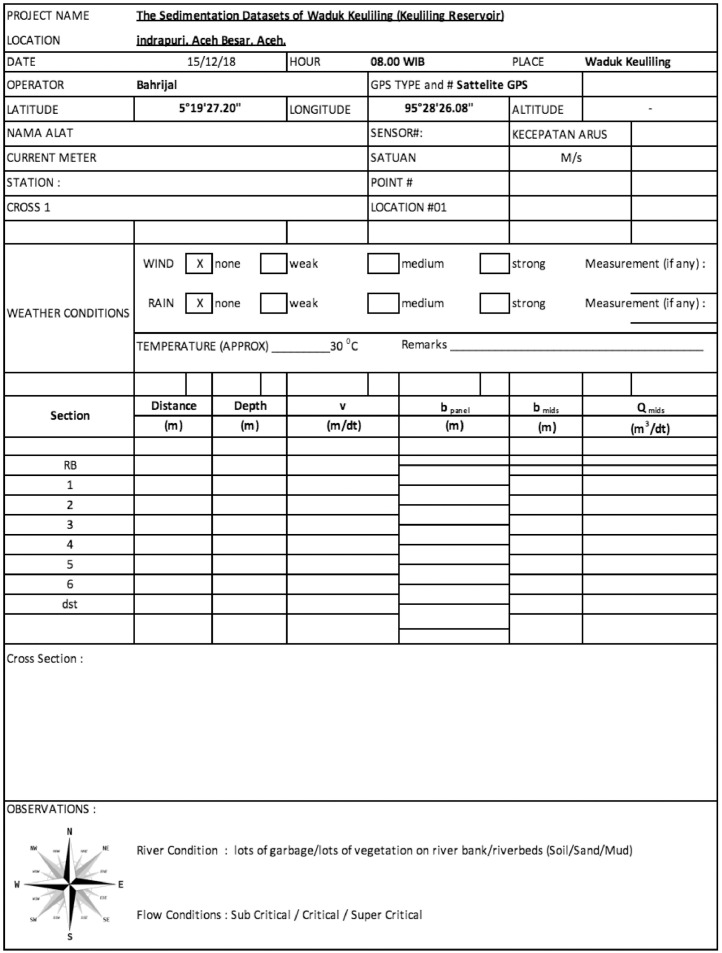
One of field data sheets

Hydrometry aims to obtain the flow velocity, river cross-section and volumetric flow rate of each channel which is the source of the Keuliling reservoir. The measurements were conducted at Alue Paku Kanan, Alue Paku Kiri, Alue Kang, Alue Keunikie, and Alue Glee Leumah. The volumetric flow rate was measured using currentmeter and the field data sheets is presented in Fig. 3. The measurement was done on April 25, 2018 . The working principle of this currentmeter is a rotating propeller due to water particles passing through it. The number of propeller turns in a certain measurement duration can generate the flow velocity measured when multiplied by the propeller calibration formula.

**Fig. 4:**
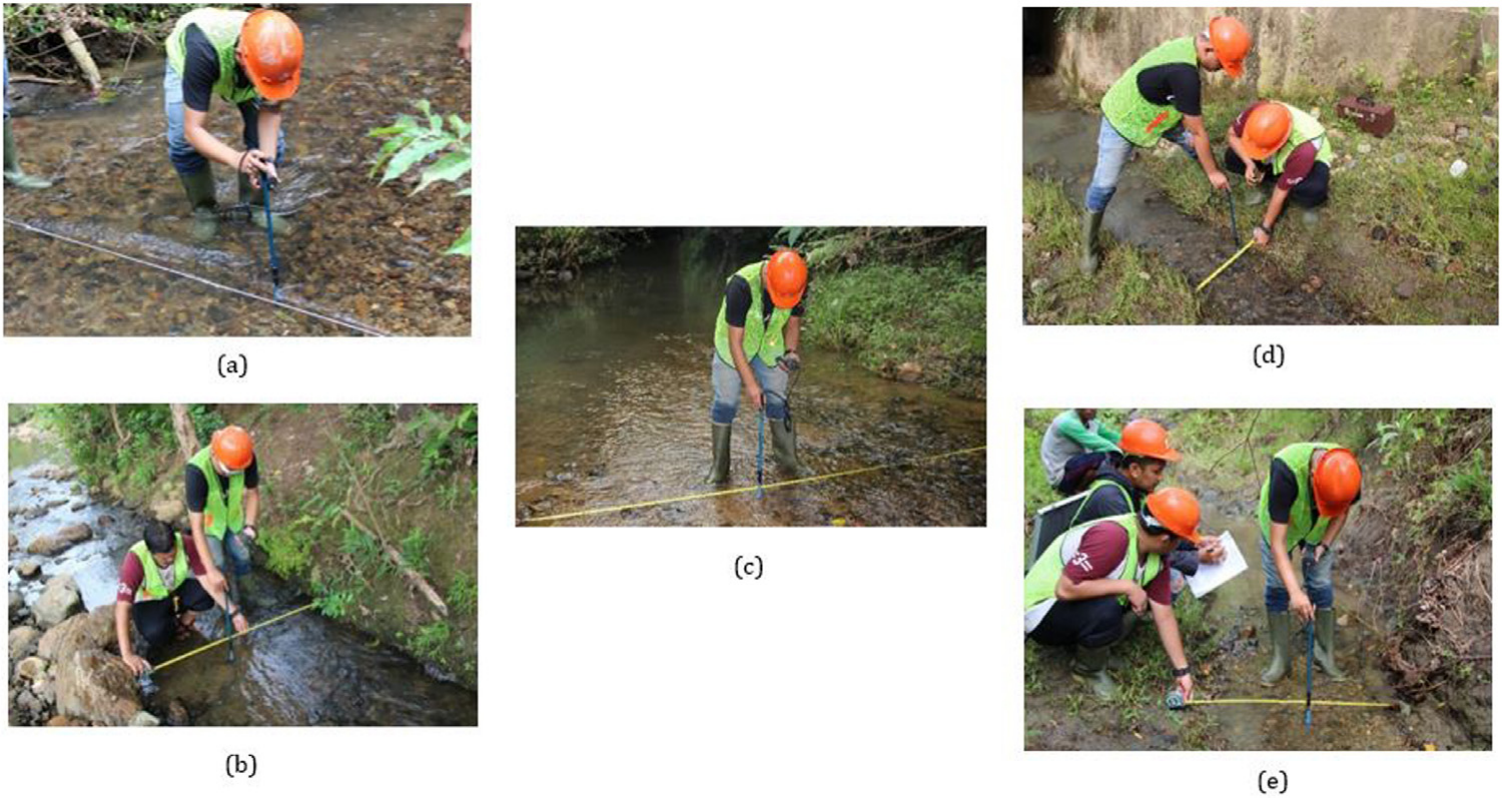
The second Hydrometric Measurement on Alue Glee Leumah (a), Alue Keunikie (b), Alue Kang (c), Alue Paku Kanan (d), Alue Paku Kiri (e)

**Fig. 5:**
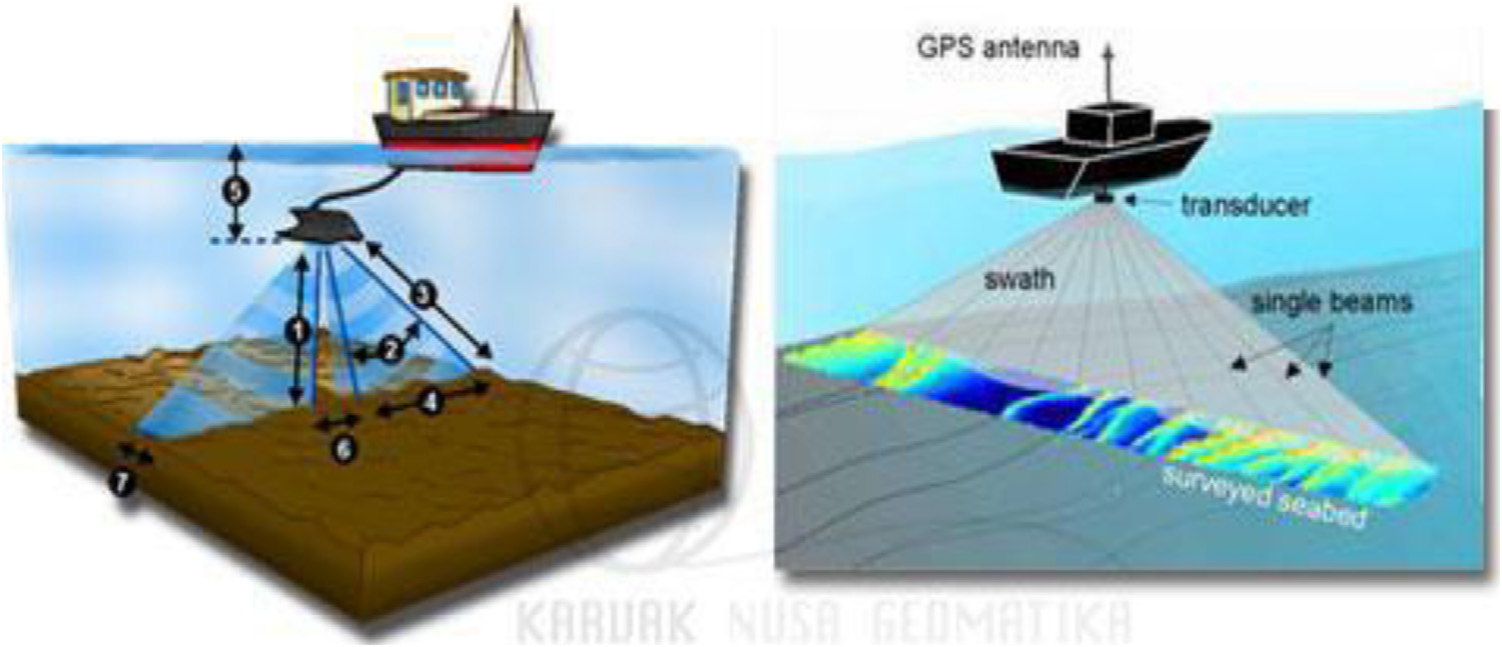
Bathymetry Survey Tools

The volumetric flow rate measurement was conducted based on the Equal Width Increment Method, by dividing the width of the river cross section into several equal parts depending on the number of samples to be taken. The vertical sampling was located in the middle of the cross section of the sampling area. The sampling was conducted using the point sample method due to the shallow and narrow flow. The results of flow velocity measurements at each Keuliling Reservoir inlet are briefly presented in Table 1. The detail of measurement results can be seen in Appendix 1.

**Table 1:** The results of flow velocity measurements

Location	Measurement 1			Measurement 2			Measurement 3		
	V	A	Q	V	A	Q	V	A	Q
Alue Paku Kanan	0.01	0.04	0.0036	0.18	0.29	0.0535	0.15	0.04	TT
Alue Paku Kiri	0.04	0.26	0.001	0.32	0.24	0.0782			
Alue Keunikie	0.05	0.03	0.0016	0.04	0.04	0.0016			
Alue Kang	0.06	0.04	0.0024	0.13	0.02	0.0024	0.1	0.03	0.0300
Alue Glee Leumah	0.07	0.25	0.0177	0.09	0.18	0.0154			

TT; Unmeasured

### 2.2 Measurement of Sediment Flows

The amount of dissolved sediment discharge can be calculated based on the measurement of volumetric flow rate and sediment sampling [5]. Suspended sediment samples were taken from a location after the volumetric flow rate measurement conducted. The equipment used for sediment sampling was USDH 48 which was also used when measuring volumetric flow rate by discharge measurements that goes straight into the river using currentmeter without boats, bridges etc. The bottle used for sample was a labelled 500 ml bottle. The bedload sediment sample was tested at the Soil Mechanics Laboratory, Civil Engineering Department, Faculty of Engineering, Muhammadiyah University, while the suspended sediment sample was examined at the Chemical Engineering Laboratory, Chemical Engineering Department, Syiah Kuala University, including the examination of specific gravity, sieve analysis and particle falling speed.

The results of specific gravity and sieve analysis are given in Table 2.

**Table 2:** Suspended Sediment Test Results

Name of Samples	TTS (Mg/L)		
	Measurement 1	Measurement 2	Measurement 3
Alue Glee Leumah	4,83	31	TT
Alue Paku Kanan	7,67	37	11
Alue Paku Kiri	22,33	13	TT
Alue Keunikie	28,00	21	TT
Alue Kang	6,17	16	9

TT: Unmeasured

### 2.3 The Measurement of Bedload Sediment

The measurement of bedload sediment transport discharge by measuring the material of the riverbed is based on the assumption that the calculation of sediment transport discharge needs to be supported by information on the bed materials [4]. In this method, riverbed material samples are taken several times at different depths of flow in the same place. However, the tool for taking riverbed material samples has a shortcoming, that it is unable to take a fine grain fraction of riverbed material. A tool used for taking the sample of riverbed materials is a grabbing sampler.

Bedload sediment sampling was taken at nine locations in the area of Keuliling Reservoir. The bedload sediments in the Keuliling Reservoir are greyed-mud which predominantly comprises Fine with a diameter of <0.075 mm as well as a few Sand (> 0.075 mm and <2.0 mm). The results of specific gravity test and sieve analysis are provided in Table 3.

**Table 3:** The Results of the Bedload Sediment Test

Number	Point	Coordinate		SGS	Atterberg Limit			Grain Size			Type of Soil
		Longitude	Latitude		LL (%)	PL (%)	PI (%)	Gravel (%)	Sand (%)	Fine (%)	
1	E	95.474050^0^	5.360618^0^	2,452	46,80	22,32	24,48	0,00	1,32	98,68	Black Mud
2	A7	95.480329^0^	5.364019^0^	2,504	34,67	20,72	13,95	0,00	8,12	91,88	gray fine sandy Mud
3	A6	95.480997^0^	5.342193^0^	2,429	66,19	34,67	31,52	0,00	5,15	94,85	Gray Mud
4	A5	95.480112^0^	5.350307^0^	2,362	45,73	26,27	19,47	0,00	5,30	94,70	dark gray mud
5	A4	95.478113^0^	5.356356^0^	2,361	57,27	25,64	31,63	0,00	6,50	93,50	Black Mud
6	A3	95.478346^0^	5.361425^0^	2,381	58,56	21,84	36,72	0,00	2,33	97,67	Black Mud
7	A2	95.475630^0^	5.363928^0^	2,498	45,87	24,12	21,76	0,00	12,38	87,62	Black Fine sandy Mud
8	A1	95.466985^0^	5.349301^0^	2,565	50,36	32,70	17,66	0,00	10,95	89,05	Black Fine sandy Mud
9	A	95.468816^0^	5.357580^0^	2,512	62,95	30,71	32,24	0,00	1,23	98,77	Gray Mud

The sediment transport can be calculated by many methods, which are Frijlink, Engelund Hansen (E-H), Leo Van Rijn, and other formula. All these formulas have a range of application in function of grain size, the slope of the bottom, the froude number, and the relative density G. The Mayer Peter Mueller (MPM) method is applied for a 0.4 ≤ d ≤ 29 mm, slopes of the bottom smaller than 0.02 and 1.25 ≤ G ≤ 4.2. After the bedload sediment test was performed as seen in Table 3, the bedload sediment calculation was conducted using the MPM method.

$$\gamma {\left(\text{Ks}/\text{Kr}\right)}^{3/2}\;R.S=0,047\left(y_{s}-y\right)d+0,25\rho^{1/3}{q_{b}}^{2/3}$$

Cy = The concentration of suspended sediment at the depth of “y”

Ca = The concentration of suspended sediment at the depth of sampling “a”

U * = Friction velocity

D = depth of flow

The results of bedload sediment calculation is shown in Table 4.

**Table 4:** The Bedload Sediment Transport Discharge

Names of Flows	Measurement 1	Measurement 2	Measurement 3
	Qbedload	Qbedload	
	(ton/day)	(ton/day)	
Glee Leumah	0.0182	1.0065	
Keunikie	0.0007	0.0009	
Kang	0.0036	0.0194	0.004
Paku Kanan	0.0138	0.0083	0.166
Paku Kiri	0.0015	0.2688	

### 2.4 The Volume of Sediment in the Keuliling Reservoir

The sediment volume in the Keuliling reservoir was calculated based on the difference of the bed elevation, as the result of the bathymetry measurement conducted in 2018, compared to the results of bathymetry measurements in 2013 and 2016 using Morphological method [1].

**Fig. 6:**
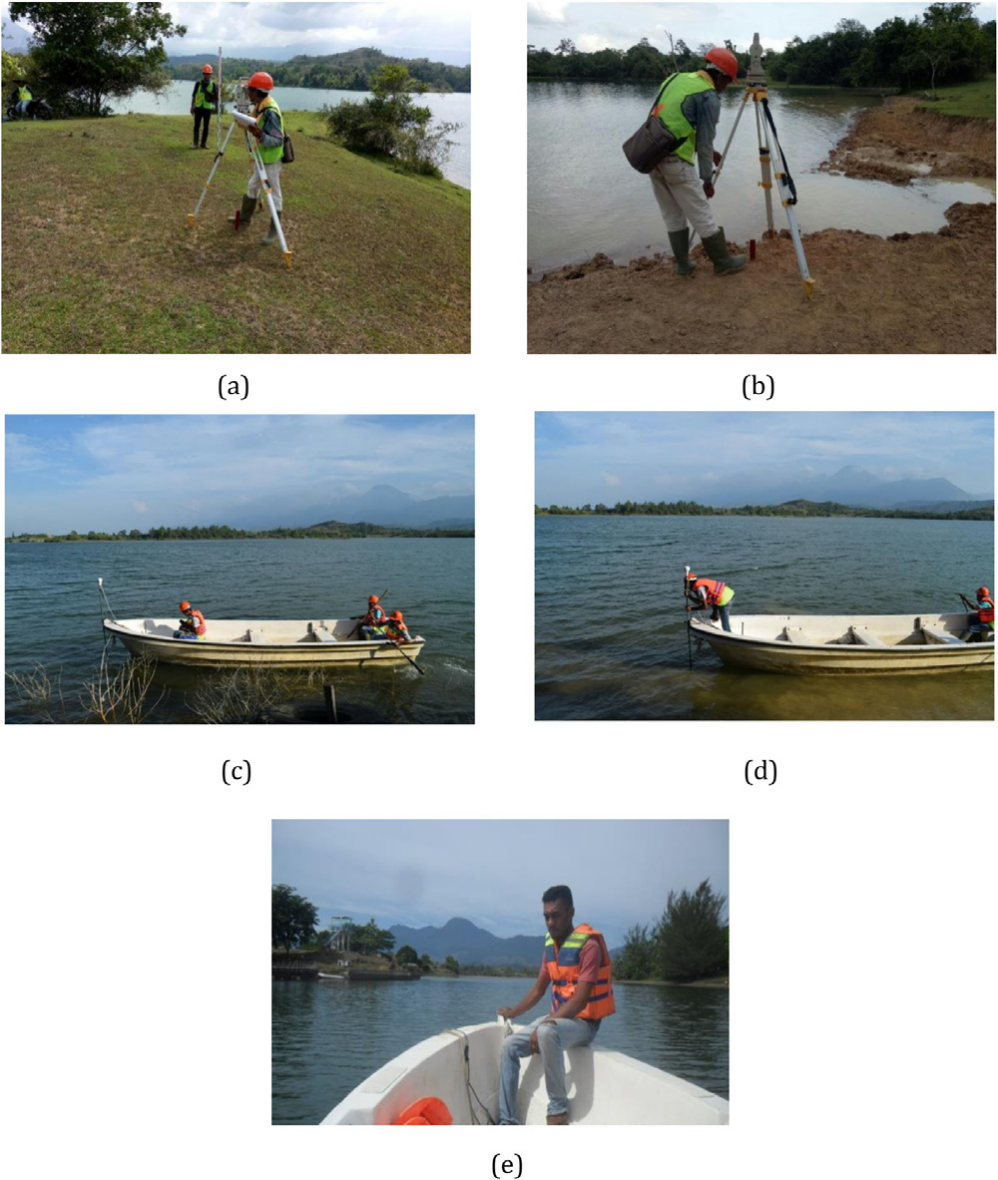
Survey Topography (a,b), Survey Bathymetry (c,d and e)

### 2.5 Bathymetry Measurement (Sounding)

Sounding is a process and activity aiming to obtain a model of the bottom surface (topographic) of the reservoir. The drawing process of the bottom of the reservoir is called a bathymetry survey (Poerbandono, 2005).

The depth measurement with echo sounder was conducted by measuring the depth of reservoir water in the predetermined sounding track. The coordinates of the points has been determined, either based on distance or time. In principle, the measurements was conducted crosswise, following the cross section. The echo-sounding technique used in the study was by measuring the depth of water by emitting regular pulses from the surface of the water and then the echo coming from the bottom of the reservoir are heard again.

The depth measurement of the Keuliling reservoir was performed using the ultrasonic wave reflection method which is perpendicularly emitted to the bottom of the water surface using the transducer (an echo sounder tool) and then the wave will be reflected back to the transducer. The formula used in the depth calculation is as follows.

(1) d=v.t

Where, d = depth (m) v = wave velocity (m / sec) t = wave propagation time (seconds)

Sounding track is the route of the ship's voyage that conducts sounding from the starting to the end point of the survey area.

The tools used in bathymetry measurements include the following.

  • Echosounder: A tool for displaying the depth
  • GPS Antenna: A tool used to obtain the coordinate position
  • Transducer: A device used to emit acoustic signals to the ocean floor to obtain the depth
  • Steel plate: for bar check/calibration.
  • Laptop: For integrating the use of GPS, transducer, and echosounder.
  • Boat: Used as a surveyor transportation and a measurement device tracing along the predetermined sounding track.

**Fig. 7:**
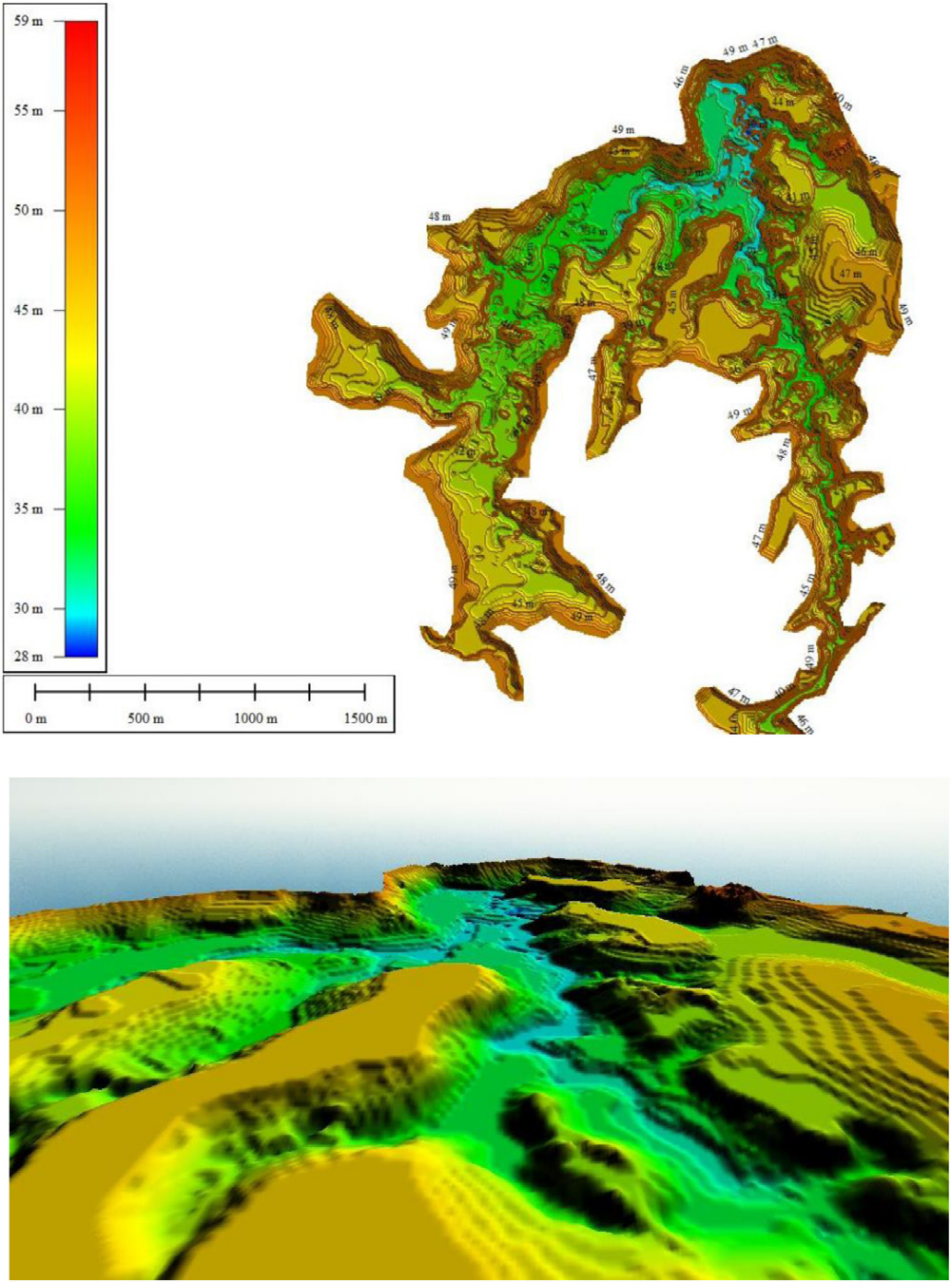
The Results of the Keuliling Reservoir Bathymetry Measurement in 2018

Topographi and Bathymetri process at Waduk Keuliling are shown in Fig. 6 which is carried out at Waduk Keuliling Area. The results of Bathymetry measurement at Keuliling reservoir are as shown in Fig. 7.

**Fig. 8:**
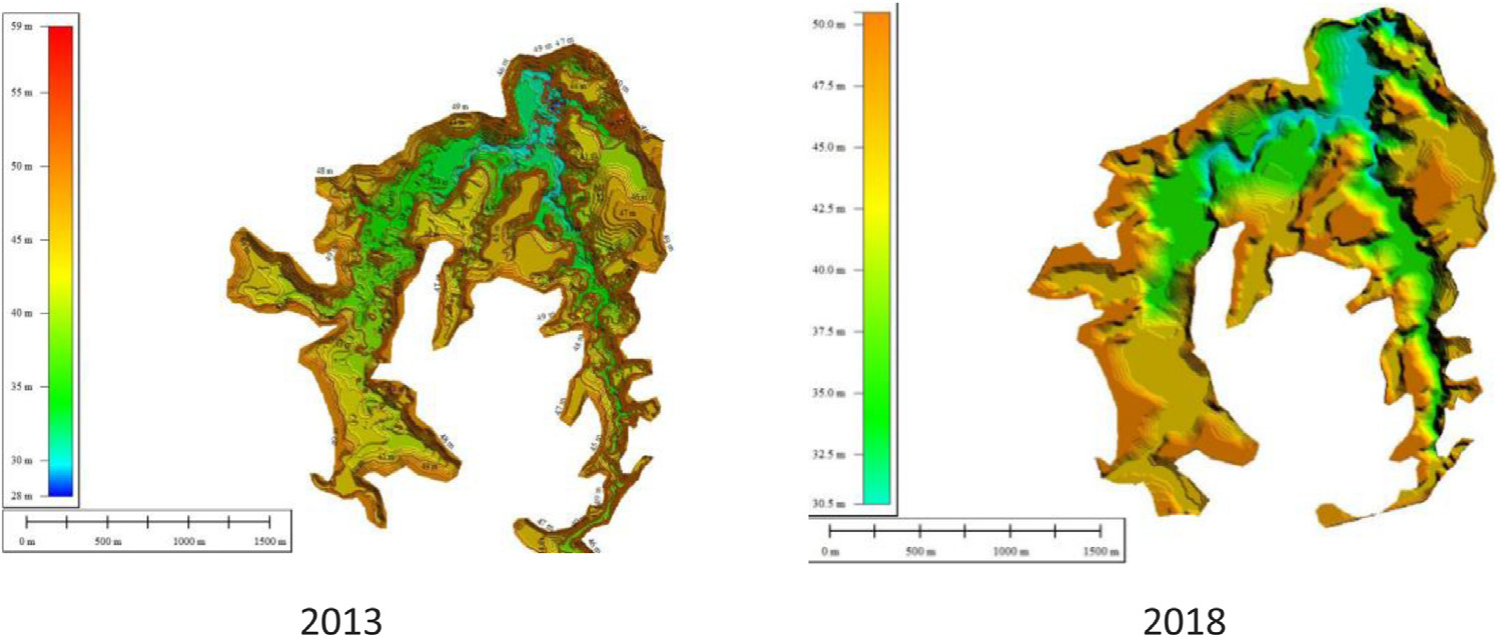
Visualitation of Keuliling Reservoir DEM year 2013 and year 2018.

**Fig. 9:**
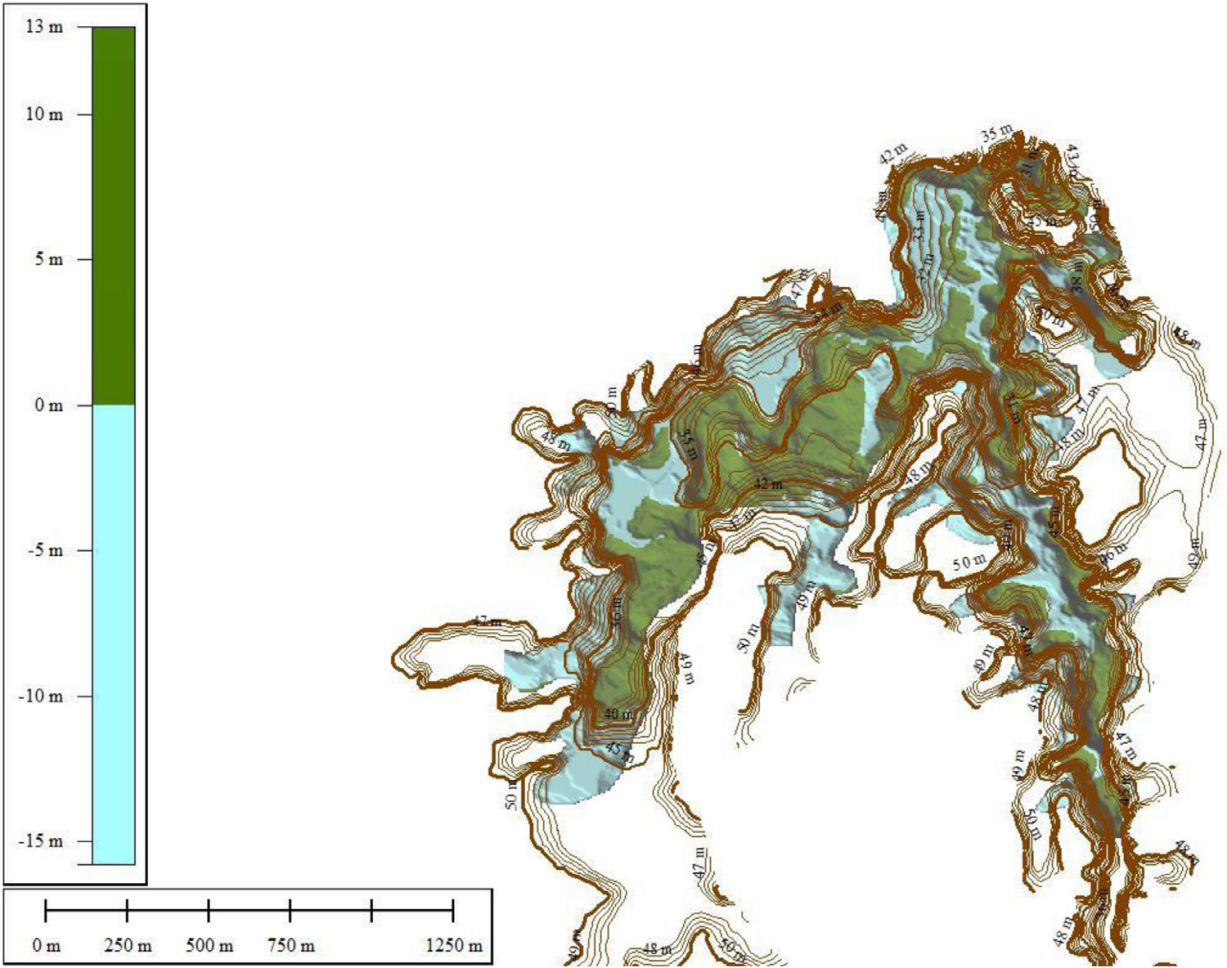
The results of the DoD in the Keuliling Reservoir area

The Evaluation of Sediments volume at Keuliling Reservoir was analyzed based on the DoD (DEM of Difference) method, or also known as Geomorphic Change Detection (GCD). The GCD is a technique where the geomorphic processes changes of erosion and sediment are deduced from repeated topographic surveys [3]. Due to the quantitative and explicit spatial results it produces, the detection of geomorphic changes is quickly becoming a tool commonly used in monitoring rivers and water bodies. The biggest problem in using this technique is the distinguishing change quantitatively due to the geomorphic process of the change caused by the "noise" and uncertainty inherent in the digital elevation model. The GCD (GCD, James et al., 2012) can also be applied to calculate the change of volume using DEM (eg Rumsby et al., 2008; Wheaton et al., 2010), or in the form of a map, where geomorphological features obtained from remote sensing, imagery or cartography (e.g. Gurnell, 1997; Surian, 1999; Hooke and Yorke, 2011).

The change volume can be calculated by applying DEM which has the same geodetic control [2] subtracted from one another to reveal the mosaic of morphological changes.

$$\delta E=Z_{2}-Z_{1}$$

where δE is the change of elevation in DEM, Z1 is the DEM surveyed earlier and Z2 is the DEM surveyed later. The total volumetric change is generated by adding up the total change (δE). The negative and positive values on the DoD map indicate erosion and sedimentation, respectively.

**Fig. 10:**
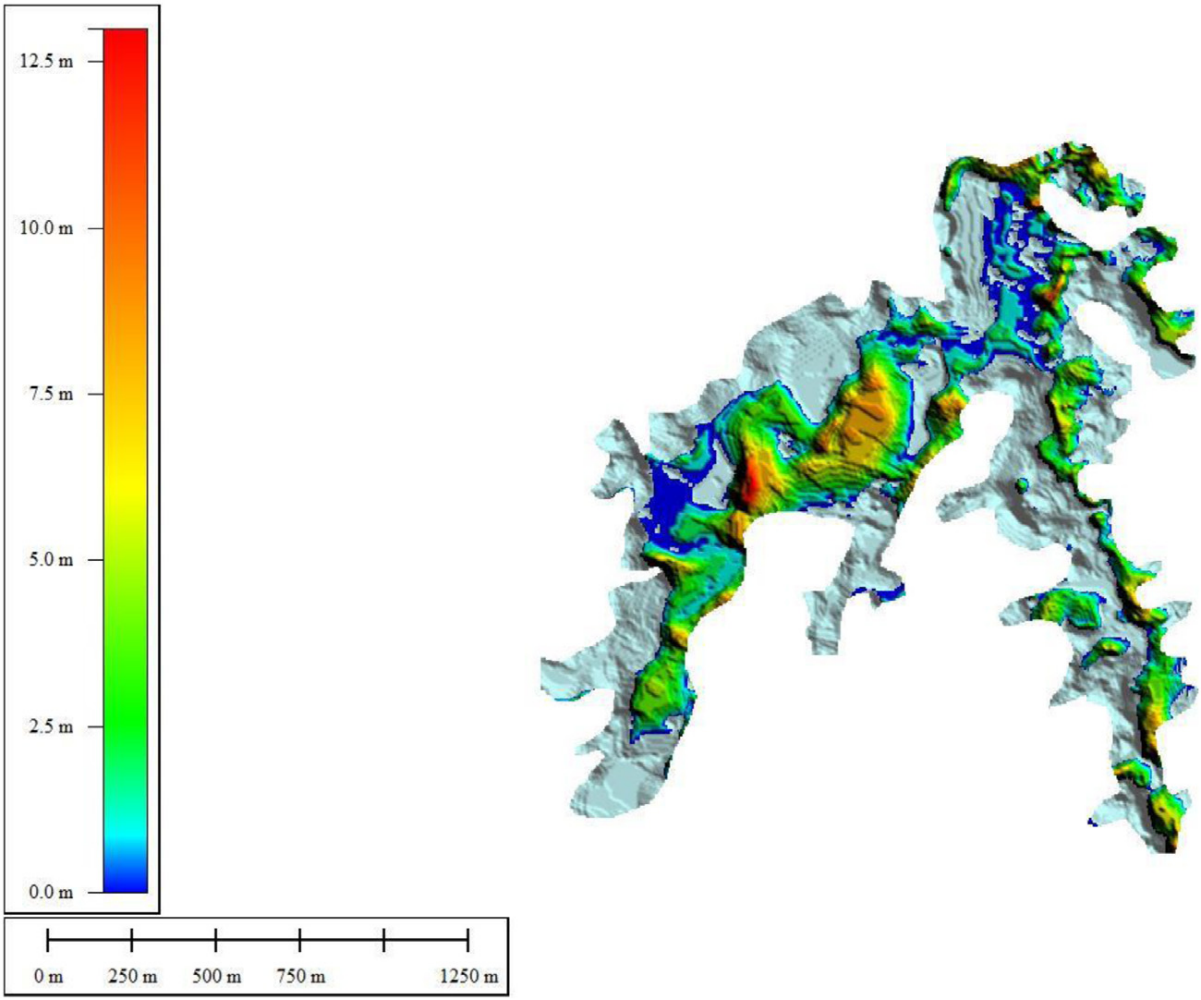
The Thickness of Sediments in the Keuliling Reservoir

To analyze the amount of sedimentation at the Keuliling reservoir based on the DoD method, the first layer was based on the 2013 topographic measurements before the Keuliling Reservoir was operated (the data was obtained from BWS Sumatra I) and the Second Layer was based on the the 2018 Keuliling Reservoir bathymetry. measurement which presented in Fig. 8. Originally Topographic and reservoir bathymetry measurement Keuliling Reservoir are point data, then convert to TIN Data, then convert to DEM with 5 m Grid Size (see Appendix 1.DEM of Keuliling Reservoir year 2013 and Year 2018). Visualitation of DEM of Keuliling Reservoir on 2013 and 2018 show in Fig. 8.

**Fig. 11:**
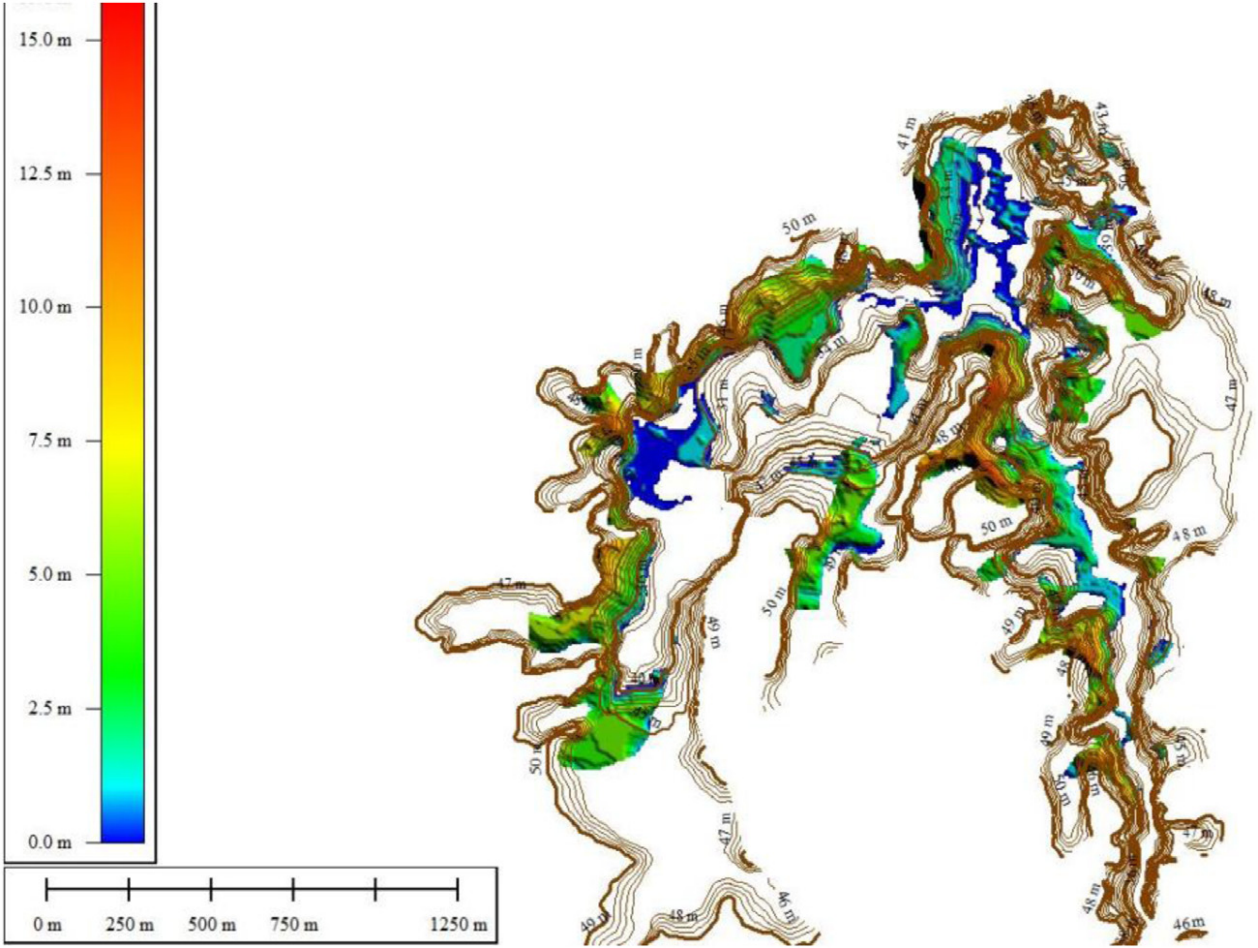
The Depth of Erosion in the Keuliling Reservoir

To limit the geomorphic changes so that it can be sufficiently objective to assess the sedimentation changes in the storage of reservoirs, DoD analysis was limited to the Keuliling Reservoir inundation area, and excluded the geomorphic changes on the land. The delineation of inundation areas in the Keuliling Reservoir is digitized in satellite imagery (See Appendix 2. DoD Boundary). The results of the DoD in the Keuliling Reservoir area (See Appendix 3. DEM of DoD Result) are Visualized in Fig. 9. The results of the DoD in the Keuliling Reservoir area are presented in Fig. 9.

The positive values indicate sedimentation, while the negative values indicate erosion. Based on the results of DOD, the analysis of sediment distribution are concluded as follows.

1 The sedimentation is concentrated in the middle of the channel, or in the deepest part of the reservoir inundation area
2 The erosion occurred at the edges bordering with the land and the shallower depths.
3 The largest sedimentation location occurs in the middle (midstream) of the inundation while it is decreasing in the downstream part, approaching the overflow building.

The sediment volume is 2,534,296.9 m^3^, while Erosion Volume is 1,846,731.6 m^3^, or the sediment Inflow for a period of five years is 687,565.3 m^3^ or approximately 137,513.06 m^3^/year.

The sedimentation thickness and The Depth of Erosion in the Keuliling Reservoir are presented in Fig. 10, Fig. 11 respectively.

## Declaration of Competing Interest

None.

## Appendix Supplementary materials

Image, application 1

Image, application 2
